# Cancer initiation and progression: an unsimplifiable complexity

**DOI:** 10.1186/1742-4682-3-37

**Published:** 2006-10-17

**Authors:** Fabio Grizzi, Antonio Di Ieva, Carlo Russo, Eldo E Frezza, Everardo Cobos, Pier Carlo Muzzio, Maurizio Chiriva-Internati

**Affiliations:** 1 Laboratories of Quantitative Medicine, Istituto Clinico Humanitas IRCCS, Via Manzoni 56, 20089 Rozzano, Milan, Italy; 2 Department of Neurosurgery, Istituto Clinico Humanitas IRCCS, Via Manzoni 56, 20089 Rozzano, Milan, Italy; 3 Department of Surgery, Texas Tech University Health Sciences Center and Southwest Cancer Treatment and Research Center, Lubbock, Texas 79430, USA; 4 Department of Microbiology & Immunology, Texas Tech University Health Sciences Center and Southwest Cancer Treatment and Research Center, Lubbock, Texas 79430, USA; 5 Division of Hematology & Oncology, Texas Tech University Health Sciences Center and Southwest Cancer Treatment and Research Center, Lubbock, Texas 79430, USA; 6 Department of Medical-Diagnostic Sciences and Special Therapies, University of Padua, Via Giustiniani 2, 35128 Padua, Italy; 7 Istituto Oncologico Veneto IRCCS, Ospedale Busonera – Via Gattamelata 64, Padua, Italy

## Abstract

**Background:**

Cancer remains one of the most complex diseases affecting humans and, despite the impressive advances that have been made in molecular and cell biology, how cancer cells progress through carcinogenesis and acquire their metastatic ability is still widely debated.

**Conclusion:**

There is no doubt that human carcinogenesis is a dynamic process that depends on a large number of *variables *and is regulated at multiple *spatial *and *temporal *scales. Viewing cancer as a system that is dynamically complex in time and space will, however, probably reveal more about its underlying behavioural characteristics. It is encouraging that mathematicians, biologists and clinicians continue to contribute together towards a common quantitative understanding of *cancer complexity*. This way of thinking may further help to clarify concepts, interpret new and old experimental data, indicate alternative experiments and categorize the acquired knowledge on the basis of the similarities and/or shared behaviours of very different tumours.

## Background

There is no doubt that cancer operates at the different levels of the hierarchical organization making up a human, and evolves through an assortment of *states *(or possible *pattern configurations*) and a number of *transitions *between two successive states [1,2].

According to the reductionist view of cancer, expressed in myriads of molecular biology-based investigations, all the information necessary for a cell to transform itself into a neoplastic cell can be attributed to changes at the genomic level. This "certainty" is based on the fact that the genome carries all of the information related to any cell process, and that any cellular transformation is due to a specific genomic change. Although this approach has offered and remains a fundamental means of generating knowledge [3-7], the accumulated information has also raised a number of fascinating questions. How many distinct regulatory circuits within each type of target cell must be disrupted in order to make it become cancerous? Is the same set of cell regulatory circuits disrupted in the cells of the disparate neoplasms arising in humans? And, if we had a complete explanation of all of the molecular reactions occurring within a living normal cell and its tumoral counterpart, would we understand that cell?

*Reductionism *seeks to interpret the wide variety of natural phenomena on the basis of the behaviour of a restricted number of basic constituents subject to simple but rigorous laws [8-10]. It has been a powerful driving force in science, as can be clearly seen from the impressive triumphs of molecular and cell biology. However, the question remains as to how to transform this molecular knowledge into an understanding of the complex phenomena existing in genes, sub-cellular entities, cells, tissues, organs, apparatuses and the body as a whole [8-18].

The need to tackle *systemic complexity *has become even more apparent since the completion of the various genome projects [19,20], which have stimulated a search for new ways of understanding the complex processes underlying cancer *initiation*, *progression *and *metastasis *[19-23].

### Initiation and progression of cancers

Cancer is today recognized as a highly heterogeneous disease: more than 100 distinct types of human cancer have been described, and various tumour subtypes can be found within specific organs [2,24]. In addition, tumours have somatic mutations and epigenetic changes, many of which are specific to the individual neoplasm [25]. It is now recognized that this genetic and phenotypical variability primarily determines the *self-progressive *growth, invasiveness and metastatic potential of neoplastic disease and its response or resistance to therapy, and it seems that the multi-level complexity of cancer explains the clinical diversity of histologically similar neoplasia [2].

Analysis of the initiation and progression of cancer cells from natural (normal) cells and the heterogeneity of a cancer population raises two intriguing questions:

• what are the properties shared by cancer and natural cells?

• to what extent are these properties shared?

**Figure 1:**
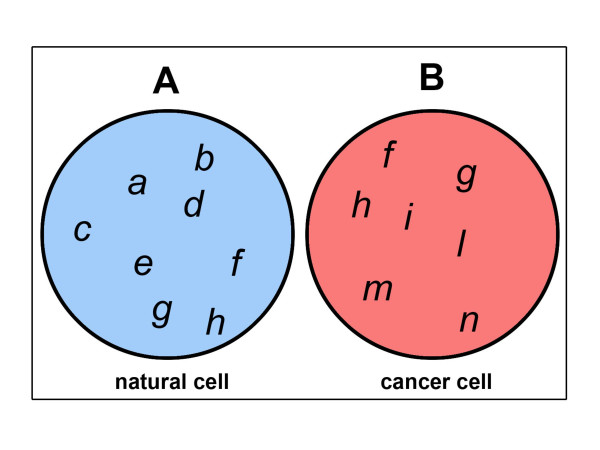
Natural cells and their tumoural counterparts can be viewed as sets of different entities and/or behaviours.

Both natural and tumoral cells are networks of sub-cellular anatomical entities organized in such a way as to perform all of the complex functions necessary to guarantee the *cell's existence*. The simplified set of real *properties *(*i.e. *characteristics) defining a natural cell can be written as:

A = {a, b, c, d, e, f, g, h}     (1)

where *A *is the set and the letters *a*, *b*, *c*...*h *indicate its *elements *or *members*: *i.e. *the cell's properties. Similarly, the simplified *set *of real properties defining a cancer cell can be written as:

B = {f, g, h, i, l, m, n}     (2)

where *B *is the set and the letters *f*, *g*, *h*...*n *indicate its *elements *or *members*. Using the graphical form suggested by Eulero-Venn [26], the two sets *A *and *B *can be drawn as in Figure 1.

**Figure 2:**
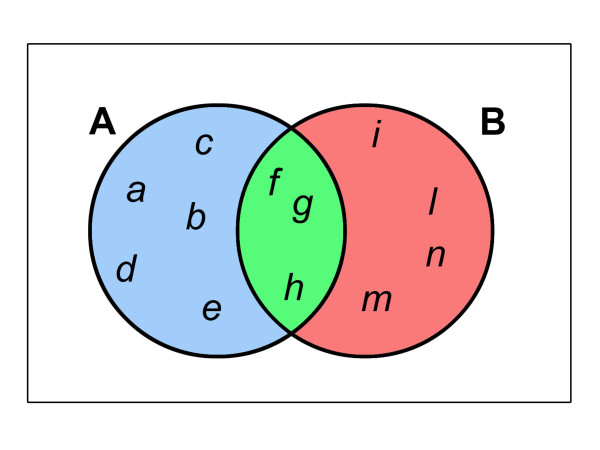
Natural and tumoural cells share a number of properties (green area), and this sharing can be schematized using set theory.

Some elements of sets *A *and *B *(*f*, *g*, *h*) are shared by natural and tumoral cells; this can be depicted graphically as the *area of intersection *between *A *and *B *(Figure 2). An important property relating the number of members of a set to their *unions *and *intersections *is:

*n*(*A*) + *n*(*B*) - *n*(*A*∩*B*) = *n*(*A*∪*B*)     (3)

In simplified terms, it can be assumed that the area of intersection is proportional to the number of properties shared by the two sets *A *and *B*.

**Figure 3:**
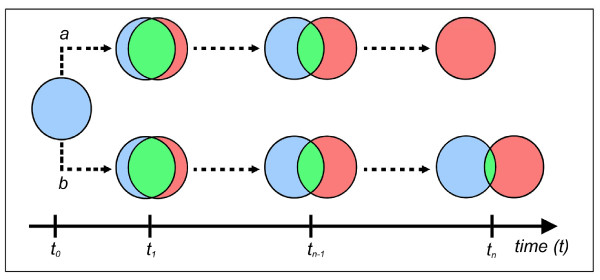
The progressive transformation of natural cells into cancer cells. This schema shows the dichotomous generation of a cancer cell as a distinct entity (*a*) or with a number of functions shared with a natural cell. The area of the intersection is proportional to the number of shared properties: a zero area indicates that the cancer cell is a wholly distinct entity, whereas its progressive but time-limited reduction leads to a cell that has a proportion of shared behaviours and a proportion of distinct properties that determine its tumoural nature *i.e. *tumour growth, invasiveness, metastatic potential and responsiveness or resistance to therapy (*b*).

On the basis of the above, it is possible to build the schema in Figure 3, which shows the hypothetical dichotomous initiation and progression of a cancer cell as: (*a*) a distinct anatomical cell entity; or (*b*) an anatomical entity that retains a number of the characteristics of a natural cell.

Natural and cancer cells are two distinct entities, each with its own pattern of properties determining its distinctive functions and/or behaviours: in other words, the generation of a final anatomical entity characterized by its own set of elements indicates that the starting set *A *and the final set *B *are distinct (Figure 3a). If we consider the area of intersection as proportional to the number of shared characteristics, the complete loss of this area over time generates a distinct entity (Figure 3a). In symbols,

*A *∩ *B *= *∅ *    (4)

In contrast, progressive but limited reduction of the area of intersection (Figure 3b) leads to a final cell that retains some of the characteristics of a normal cell but has a number of distinct properties that are responsible for its *tumoral nature i.e. *tumour growth, invasiveness, metastatic potential and responsiveness or resistance to therapy. Consequently, the shared properties that are retained between the two sets A and B may result in collateral effects or toxicity during therapeutic regimens.

It is interesting to note that the *asynchrony *and *self-progression *of a cancer cell population suggest that the extent to which each neoplastic cell shares the properties of a natural cell may differ in *time *and in *space*. It is also worth pointing out that the differences or similarities between natural and cancer cells may involve both *qualitative *and *quantitative *aspects.

Recently, the development of recurrent cancers and second primary tumours (SPTs) has been explained by the concept of "field cancerization" [28]. In the original paper by Slaughter et al., field cancerization was defined as follows: (*a*) oral cancer develops in multi-focal areas of precancerous change, (*b*) histologically abnormal tissue surrounds the tumours, (*c*) oral cancer often consists of multiple independent lesions that sometimes coalesce, and (*d*) the persistence of abnormal tissue after surgery may explain SPTs and local recurrences [29]. Genetic analyses have been performed to substantiate the observation of Slaughter et al. that tissue adjacent to a tumour can be aberrant. Importantly, many investigators have found cancer-associated genetic alterations in tumour-adjacent "macroscopically normal" tissue and its margins [27,28]. Fields with genetically altered cells can be large (up to 7 cm in diameter). These lesions may not be apparent on histopathological investigation but can be detected by molecular analyses for phenotypic or genetic alterations associated with carcinogenesis such as p53 gene mutations, integrated viral DNA, loss of heterozygosity and microsatellite instability. Field cancerization has been described for lung, oesophagus, vulva, cervix, anus, colon, breast, bladder and skin in addition to the oral cavity, pharynx and larynx [27,28].

In the experimental sciences, observed patterns can often be conceptualized as *macro-scale *manifestations of *micro-scale *processes. However, in many cases, a more typical situation involves observed patterns or system states that are created or influenced by *multiple processes *and *controls *[30]. Cancer is determined by a number of processes and controls operating over much broader scales, and by factors such as *structural controls *that may operate at scales ranging from molecular to environmental [2]. This *multiple scale causality *not only recognizes multiple processes and controls acting at multiple scales but, in contrast to a strictly reductionist approach, may also recognize that relevant "first principles" may reside at scales other than the smallest micro-scales. In other words, the observed phenomenon at each scale has structural and behavioural properties that do not exist at lower or higher organizational levels [2].

Furthermore, it is necessary to consider that the complex *environment *in which a normal or cancer cell is embedded determines a different *cell-environment interchange *of *matter*, *energy *and *information*, thus inducing continuous qualitative and/or quantitative changes in the shared properties and/or behaviours. The milieu of environmental effects responsible for the genesis of neoplastic precursor cells is often associated with autoimmune or chronic inflammatory reactions induced by biological agents, endogenous and exogenous chemicals, and physical agents such as heat, radiation and foreign bodies [31].

### Conclusive key-points

On the basis of the above, there is no doubt that human carcinogenesis is a complex dynamic process that depends on a large number of *variables *and is regulated at multiple *spatial *and *temporal *scales, thus making it one of the most intricate phenomena in biology [2].

In mathematical terms, carcinogenesis is a *non-linear *process, the behaviour of which does not follow clearly predictable and repeatable pathways. In *linear systems*, the behaviour of a system changes linearly in response to an environmental factor that influences it. In contrast, the behaviour of non-linear complex systems may be perceived as *surprising *and *unpredictable *[32]. Periods of inactivity may be punctuated by sudden change, apparent patterns of behaviour may disappear and new patterns may unexpectedly emerge [2]. Moreover, non-linear systems do not react proportionally to the magnitude of their *inputs*, and depend on their *initial conditions*, *i.e. *small changes in the initial conditions may generate very different end-points. These characteristics are frequently highlighted by the frequency with which differences in progression or therapeutic response are seen in the same tumour type, and by the fact that cancer morphology does not always reveal a similar underlying biology. It is also necessary to emphasize that cancer does not conform to simple mathematical principles: the irregular mode of carcinogenesis, erratic tumour growth, variable response to tumoricidal treatments, and poorly understood metastatic patterns constitute highly variable clinical behaviours.

After the complete resection of a carcinoma preserving part of the organ in which it developed, microscopically normal but genetically altered epithelium may remain in situ and acquire additional mutations or epigenetic alterations that can initiate the development of a second tumor of the same or a different histological type, representing an *in situ *recurrence. Local recurrences develop at the site of the primary surgery/surgical scar (scar recurrences) or at some distance from this location in the residual organ that remained in situ after resection (*in situ *recurrences). Discrimination between these two types of local recurrence is important as their different prognoses point to differences in their pathogenesis.

Recently, Tabor et al. [33] have shown that in a proportion of patients with primary head and neck tumours, the primary tumour and the SPT develop from a single contiguous genetically-altered field and thus have a common clonal origin. In other patients, the first and second primary tumours develop independently from genetically unrelated fields [33]. The picture that emerges is that in the second primary tumor patients, large areas of the normal mucosa have been replaced by one or more monoclonal cell populations [33].

In conclusion, the above reflections have led us to think that:

(1) Cancer is a highly complex disease in time and in space [2,34-37].

(2) Cancer is a hierarchical system. The decisive step in carcinogenesis is the result of an irreversible qualitative change in one or more of the genetic characteristics of cancer cells. Although this modification governs the transformation of normal human cells into malignant cancer cells, it may or may not lead to visible changes in their cytological or histological structures [2]. This can be explained using the concept of *emergence*, which defines humans as complex systems consisting of different anatomical entities that are interconnected at many organizational levels (a hierarchical system), have various degrees of complexity, and are governed by specific laws that only operate at a particular level [2].

(3) First proposed for oral cancer by Slaughter *et al. *[29], field cancerization describes clinically occult multifocal precancerous lesions of the epithelium within an anatomical region exposed to the same carcinogen(s) [e.g. cigarette smoking, human papillomavirus (HPV) infection]. Current adjuvant treatment modalities are effective in eradicating tumor cells, but these may not be the treatment of choice for relatively large fields of precancerous cells. Moreover, the shared properties retained between the two sets A and B (Figure 3) may result in collateral effects or toxicity during the therapeutic regimens.

(4) Although the alterations usually occur at a characteristic stage of tumour advancement, experimental evidence indicates that the ongoing accumulation of changes is more important than their order of occurrence in the course of cancer [2]. In physical terms, it is true that alterations in one parameter (*i.e. *chromosomal changes, DNA changes, specific gene changes or mitochondrial changes) are not necessarily associated with the loss of stability of a system, and it is also true that an *unstable system *is more sensitive to small changes in parameters (*i.e. *its state is more easily modifiable). In biological terms, a growing network of cancer-susceptibility genes is formed as the neoplasm advances [2]. The human genome is typically so stable that the many genetic alterations required for cancer to develop cannot accumulate unless the rate of mutation increases to the point of making it genetically unstable [2,38].

(5) Considering cancer as a *robust system *[*i.e. *the ability of a living system to maintain performance (*phenotypic stability*) in the face of perturbations arising from environmental changes, stochastic events (or *intracellular noise*) and genetic variations] would provide us with a framework for future research strategies, and future cancer therapies may be judged on their ability to help control the robustness of tumours [2,39].

(6) Modelling the growth and development of human tumours using mathematics and biological data is a burgeoning area of cancer research. Mathematical methods and their derivatives have proved to be possible and practical in oncology [40], but the current models are often simplifications that ignore vast amounts of knowledge: for example, most metabolic models seem to regard a cell as a bag of enzymes, neglecting its spatial heterogeneity and compartmentalisation [2]. Furthermore, most models struggle to resolve the 10–12 order-of-magnitude span of the timescales of systemic events, be they molecular (ion channel gating: 10^-6 ^s), cellular (mitosis: 10^2^–10^3 ^s) or physiological (cancer progression: 10^8 ^s) [2].

Viewing cancer as a system that is dynamically complex in time and space will, however, probably reveal more about its underlying behavioural characteristics. It is encouraging that mathematicians, biologists and clinicians continue to contribute together towards a common quantitative understanding of *cancer complexity *[1,2,40-43]. This way of thinking may further help to clarify concepts, interpret new and old experimental data, indicate alternative experiments and categorize the acquired knowledge on the basis of the similarities and/or shared behaviours of very different tumours.

## Competing interests

The author(s) declare that they have no competing interests.

## Authors' contributions

FG, MCI conceived, coordinated and designed the study, and drafted the manuscript; ADI, CR, EEF, EC, PCM participated in drafting the manuscript. All of the authors have read and approved the final manuscript.
